# Secretory products from epicardial adipose tissue induce adverse myocardial remodeling after myocardial infarction by promoting reactive oxygen species accumulation

**DOI:** 10.1038/s41419-021-04111-x

**Published:** 2021-09-13

**Authors:** Shuang Hao, Xin Sui, Jing Wang, Jingchao Zhang, Yu Pei, Longhui Guo, Zhenxing Liang

**Affiliations:** 1 Department of Cardiac Surgery, The First Affiliated Hospital of Zhengzhou University, 450000 Zhengzhou, China; 2 Department of Oncology, The First Affiliated Hospital of Zhengzhou University, 450000 Zhengzhou, China

**Keywords:** Cell biology, Molecular biology

## Abstract

Adverse myocardial remodeling, manifesting pathologically as myocardial hypertrophy and fibrosis, often follows myocardial infarction (MI) and results in cardiac dysfunction. In this study, an obvious epicardial adipose tissue (EAT) was observed in the rat model of MI and the EAT weights were positively correlated with cardiomyocyte size and myocardial fibrosis areas in the MI 2- and 4-week groups. Then, rat cardiomyocyte cell line H9C2 and primary rat cardiac fibroblasts were cultured in conditioned media generated from EAT of rats in the MI 4-week group (EAT-CM). Functionally, EAT-CM enlarged the cell surface area of H9C2 cells and reinforced cardiac fibroblast activation into myofibroblasts by elevating intracellular reactive oxygen species (ROS) levels. Mechanistically, miR-134-5p was upregulated by EAT-CM in both H9C2 cells and primary rat cardiac fibroblasts. miR-134-5p knockdown promoted histone H3K14 acetylation of manganese superoxide dismutase and catalase by upregulating lysine acetyltransferase 7 expression, thereby decreasing ROS level. An in vivo study showed that miR-134-5p knockdown limited adverse myocardial remodeling in the rat model of MI, manifesting as alleviation of cardiomyocyte hypertrophy and fibrosis. In general, our study clarified a new pathological mechanism involving an EAT/miRNA axis that explains the adverse myocardial remodeling occurring after MI.

## Introduction

Myocardial infarction (MI) is a severe coronary artery-related disease with high morbidity and mortality worldwide [1]. Post MI, the infarcted heart occurs adverse myocardial remodeling, manifesting as cardiac hypertrophy and fibrosis, which impairs heart function and eventually leads to heart failure [2]. Recent studies have linked the development of adverse myocardial remodeling after MI to epicardial adipose tissue (EAT). EAT is located between the myocardium and the visceral pericardium and accounts for 20% of the weight of the human heart [3]. Since no fascia separates the EAT from the myocardium, EAT can directly regulate myocardial function by secreting bioactive molecules [4]. After MI, a disorder in the levels of secretory products from EAT is observed and the EAT thickness is increased in association with myocardial fibrosis development [5]. In a rat model of MI, the removal of EAT after MI decreases myocardial infarction size and improves myocardial function [6]. These findings suggest a potential role for EAT and its secretory products in adverse myocardial remodeling after MI.

The pathogenesis of MI is closely associated with mitochondrial oxidative stress [7]. During MI progression, the depletion of cellular ATP induced by ischemia results in mitochondrial dysfunction that facilitates the generation of reactive oxygen species (ROS) [8]. The accumulated ROS activates a series of hypertrophy signaling kinases, thereby contributing to myocardial hypertrophy, while also promoting myocardial fibrosis by activating matrix metalloproteinases [9]. In the preliminary experiment, we found that compared with rats in the control group, rats with MI exhibited evident hypertrophy of the left ventricular myocardium (Supplementary Fig. 1A), myocardial fibrosis (Supplementary Fig. 1B), with concomitant ROS accumulation (Supplementary Fig. 1C). Notably, the anti-oxidant agent probucol can effectively limit adverse myocardial remodeling in a rat model of MI by scavenging excessive ROS [10]. Interestingly, the research of Blumensatt et al. [11] showed that incubation of primary rat cardiomyocytes in conditioned media generated from the EAT of patients with type 2 diabetes impaired mitochondrial respiration. Therefore, we hypothesize that EAT and its secretory products exacerbate mitochondrial dysfunction, thereby intensifying the ROS-mediated adverse myocardial remodeling occurring after MI.

Mitochondrial function is now known to be subject to regulation by microRNAs (miRNAs), which are noncoding RNAs about 20 nucleotides in length that regulate target gene expression at the post-transcriptional level [12]. For instance, miR-762 overexpression increases ROS level and decreases ATP generation in anoxia/reoxygenation-treated cardiomyocytes by targeting NADH dehydrogenase subunit 2 [13]. Other research has shown an upregulation of miR-143 in cardiomyocytes and subsequent suppression of insulin action in response to the activin A secreted by EAT from patients with type 2 diabetes [14], indicating the possible regulation of miRNA expression in recipient cells by secretory products from EAT. However, whether EAT and its secretory products can regulate ROS-mediated adverse myocardial remodeling after MI by affecting specific miRNAs level remain unclear.

In this study, we used a combination of a rat model of MI, the rat cardiomyocyte cell line H9C2, and primary rat cardiac fibroblasts, to evaluate the potential role of EAT in adverse myocardial remodeling after MI in vivo and in vitro. We also sought to identify specific miRNAs that could mediate the regulatory effect of EAT on myocardial hypertrophy and fibrosis to further our understanding of the pathogenesis of adverse myocardial remodeling after MI and to provide new targets for its prevention and treatment.

## Materials and methods

### Establishment of MI in rats

Male Sprague-Dawley (SD) rats (8–10 weeks old) were purchased from Beijing Vital River Laboratory Animal Technology Co., Ltd (China). A rat model of MI was established as previously described [15]. Briefly, the rat was anesthetized, the heart was exposed via a thoracotomy at the level of the left third and fourth intercostal spaces, and the proximal left anterior descending coronary artery (LAD) was ligated using a 7–0 Prolene suture (Qingdao Nesco Medical Co., Ltd., China). Normal rats were used as the control group (*n* = 10). At 1 (*n* = 13), 2 (*n* = 10), and 4 (*n* = 15) weeks after MI, the rats were euthanized and the cardiac tissues were collected.

To evaluate the effect of miR-134-5p on the adverse myocardial remodeling in the rat model of MI, adeno-associated virus (AAV)-sh-miR-134-5p and its negative control (AAV-NC) were synthesized by Han Heng Biotechnology Co., Ltd (Shanghai, China). The SD rats were divided into sham (*n* = 10), MI + AAV-sh-miR-134-5p (*n* = 10), and MI + AAV-NC (*n* = 10) groups. The rat model of MI was established using LAD ligation as described above. Rats that underwent the same procedure without ligation were used as the sham group. One week after LAD ligation, 100 µl (2 × 10^12^ v.g.) AAV-NC and AAV-sh-miR-134-5p were intravenously injected into the rats of the MI + AAV-NC and MI + AAV-sh-miR-134-5p groups, respectively. Four weeks after LAD ligation, all rats were euthanized, and the left ventricular tissues were collected. All animal experimental procedures were performed with the approval of the Animal Ethics Committee of The First Affiliated Hospital of Zhengzhou University.

### Preparation of conditioned media from EAT (EAT-CM)

EAT-CM was prepared from the EAT of rats in the MI 4-week group as described previously [11]. After washing with PBS, the EAT was cut into 10 mg explant pieces and washed three times with PBS. The EAT explants were centrifuged at 1, 200 rpm for 1 min, and the sedimented EAT explants were cultured in DMEM-F12 medium containing 10% FBS, 33 µmol/l biotin, 17 µmol/l d-pantothenate, and antibiotics at 37 °C. On the second day, the EAT explants were harvested and cultured in serum-free DMEM-F12 medium containing 33 µmol/l biotin, 17 µmol/l D-pantothenate, and antibiotics at a concentration of 100 mg EAT explants/ml medium. On the third day, the EAT explants were removed and the EAT-CM was collected for subsequent experiments.

### Additional methods

Detailed methodology is described in the Supplementary material.

## Results

### The occurrence of myocardial hypertrophy and fibrosis in the rat model of MI

**Fig. 1:**
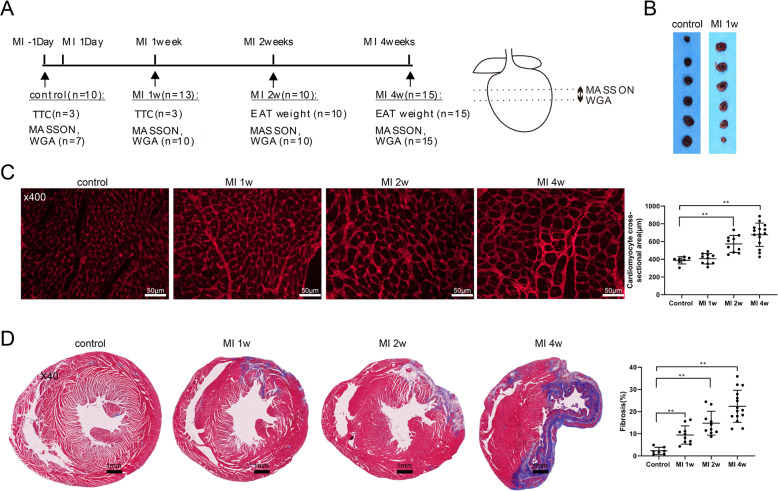
Adverse myocardial remodeling in the rat model of myocardial infarction (MI). **A** Left: experimental protocols of control (*n* = 10), MI 1-week (*n* = 13), MI 2-week (*n* = 10), and MI 4-week (*n* = 15) groups. Right: wheat germ agglutinin (WGA) staining and Masson’s trichrome staining were carried out with the tissue at the location indicated by the dotted line. **B** Representative cardiac slices of rats with infarcts stained by 2,3,5-triphenyl tetrazolium chloride (TTC) in the control and MI 1-week groups. **C** Cardiomyocyte cross-sectional areas were examined using WGA staining (scale bar = 50 µm). **D** Myocardial fibrosis was examined using Masson’s trichrome staining (scale bar = 1 mm). **P < 0.01.

The rat model of MI was established by permanent ligation of the LAD and the experimental protocol is presented in Fig. 1A. Compared with the rats in the control group, rats in the MI 1-week group exhibited an increased cardiac infarct size (Fig. 1B), thereby confirming the successful induction of experimental MI. Assessment of the cardiomyocyte size and myocardial fibrosis by wheat germ agglutinin (WGA) staining and Masson’s trichrome staining, respectively, revealed that the rats in the MI group exhibited enlarged cardiomyocyte cross-sectional areas and the areas of myocardial fibrosis (Fig. 1C, D). These findings indicated the occurrence of adverse myocardial remodeling in rats with MI, manifesting as myocardial hypertrophy and fibrosis.

### EAT was associated with adverse myocardial remodeling after MI

**Fig. 2:**
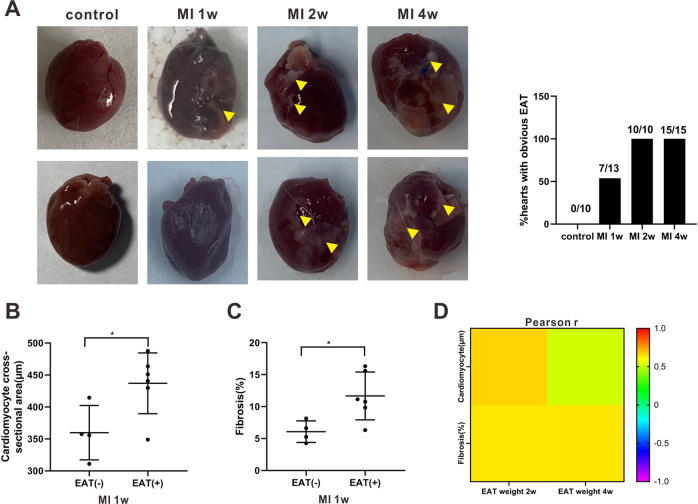
The correlation between epicardial adipose tissue (EAT) and adverse myocardial remodeling. **A** Left: Representative images of hearts of rats in the control, MI 1-week, MI 2-week, and MI 4-week groups. Triangles: obvious EAT. Right: The ratio of hearts with obvious EAT in each group. The (**B**) cardiomyocyte cross-sectional areas and (**C**) myocardial fibrosis areas in the rats with (+) or without (−) EAT in MI 1-week group. *P < 0.05. **D** Pearson’s correlation coefficient (*r*) of EAT weights and cardiomyocyte size/myocardial fibrosis areas in rats in the MI 2-week and MI 4-week groups.

Then, the correlation between EAT and adverse myocardial remodeling in rats with MI was investigated. As shown in Fig. 2A, an obvious EAT was observed in about half of the rats in the MI 1-week group and in all rats in the MI 2-week and 4-week groups. The rats in the MI 1-week group with obvious EAT exhibited larger cardiomyocyte cross-sectional areas and more severe myocardial fibrosis compared with the rats in the same group that did not show obvious EAT (Fig. 2B, C). Correlation analysis confirmed a positive correlation between EAT weights and both the cardiomyocyte size and the myocardial fibrosis areas in the rats in the MI 2-week and 4-week groups (Fig. 2D and Supplementary Fig. 1D–G).

### Secretory products from EAT promoted cardiomyocyte hypertrophy and cardiac fibroblast activation by elevating the ROS level

**Fig. 3:**
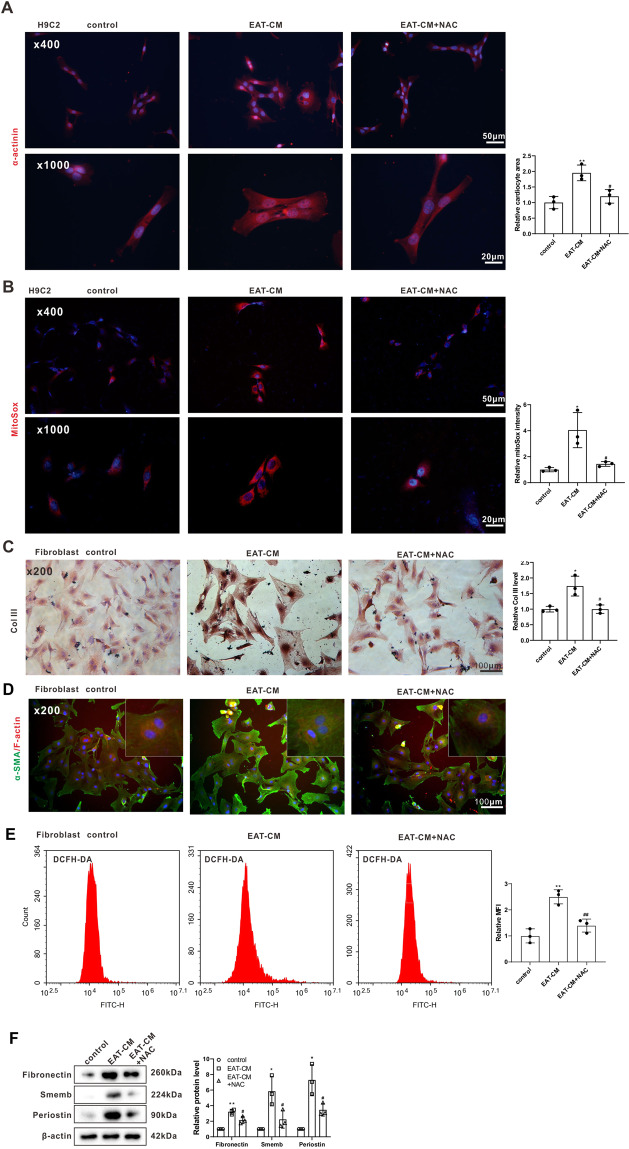
Secretory products from EAT promoted cardiomyocyte hypertrophy and cardiac fibroblast activation by elevating ROS levels. Rat cardiomyocyte cell line H9C2 and primary rat cardiac fibroblasts were cultured in DMEM-F12 medium, EAT conditioned media (EAT-CM), or EAT-CM + *N*-acetyl-cysteine (NAC, 2 mM) for 72 h. **A** Immunofluorescent staining for α-actinin in H9C2 cells. **B** MitoSox fluorescence probe was used to observe the mitochondrial superoxide content in H9C2 cells. Cell nuclei were counterstained with DAPI. **C** Immunohistochemical staining for collagen III (CoI III) in primary rat cardiac fibroblasts (scale bar = 100 µm). **D** Immunofluorescent staining for alpha-smooth muscle actin (α-SMA) (green)/filamentous actin (F-actin) (red) in primary rat cardiac fibroblasts (scale bar = 100 µm). Cell nuclei were counterstained with DAPI. **E** The intracellular ROS level in primary rat cardiac fibroblasts was analyzed by flow cytometry using a DCFH-DA fluorescence probe. MFI: mean fluorescence intensity. **F** The protein levels of fibronection, smemb, and periostin in primary rat cardiac fibroblasts were measured by western blot. β-actin was used as an internal control. *P < 0.05, **P < 0.01 vs H9C2 or primary rat cardiac fibroblasts cultured in DMEM-F12 medium (control); ^#^*P* < 0.05, ^##^*P* < 0.01 vs H9C2 or primary rat cardiac fibroblasts cultured in EAT-CM (EAT-CM).

To further explore the effect of EAT on myocardial hypertrophy and fibrosis, rat cardiomyocyte cell line H9C2 and primary rat cardiac fibroblasts were cultured in EAT-CM for 72 h, and the cardiocyte area and cardiac fibroblast activation were examined, respectively. Immunofluorescent staining for α-actinin, a cardiomyocyte-specific marker [16], showed that exposure to EAT-CM significantly increased the cell surface area of the H9C2 cells (Fig. 3A). Immunofluorescent staining for alpha-smooth muscle actin (α-SMA) (a myofibroblast marker [17])/filamentous actin (F-actin) and immunohistochemical staining for CoI III (a type of collagen produced by myofibroblasts [18]) showed that exposure to EAT-CM reinforced the cardiac fibroblast activation into myofibroblasts (Fig. 3C, D). The elevations in the protein levels of fibronectin, smemb, and periostin in primary rat cardiac fibroblasts exposure to EAT-CM also supported this conclusion (Fig. 3F). Besides, the mitochondrial superoxide content in H9C2 cells and the intracellular ROS level in primary rat cardiac fibroblasts were detected using MitoSOX and DCFH-DA fluorescent probes, respectively. As shown in Fig. 3B, MitoSox fluorescence intensity increased in H9C2 cells cultured in EAT-CM compared to H9C2 cells cultured in DMEM-F12 medium. Similarly, primary rat cardiac fibroblasts cultured in EAT-CM exhibited enhanced DCFH-DA fluorescence intensity compared to primary rat cardiac fibroblasts cultured in DMEM-F12 medium (Fig. 3E). To determine whether EAT-CM promoted cardiomyocyte hypertrophy and cardiac fibroblast activation by affecting mitochondrial function, H9C2 cells and primary rat cardiac fibroblasts were cultured in EAT-CM supplemented with *N*-acetyl-cysteine (NAC), a ROS scavenger. As shown in Fig. 3A, NAC supplementation significantly suppressed the cell surface area expansion of H9C2 cells due to EAT-CM culture. Meanwhile, the incorporation of α-SMA into F-actin stress fibers and protein levels of CoI III, fibronectin, smemb, and periostin in primary rat cardiac fibroblasts cultured in EAT-CM + NAC were also reduced compared to primary rat cardiac fibroblasts cultured in EAT-CM (Fig. 3C, D). These findings confirmed that secretory products from EAT promoted cardiomyocyte hypertrophy and cardiac fibroblast activation by upregulating the ROS level.

### Cardiomyocyte hypertrophy and cardiac fibroblast activation induced by secretory products from EAT involved miR-134-5p regulation of intracellular ROS levels

**Fig. 4:**
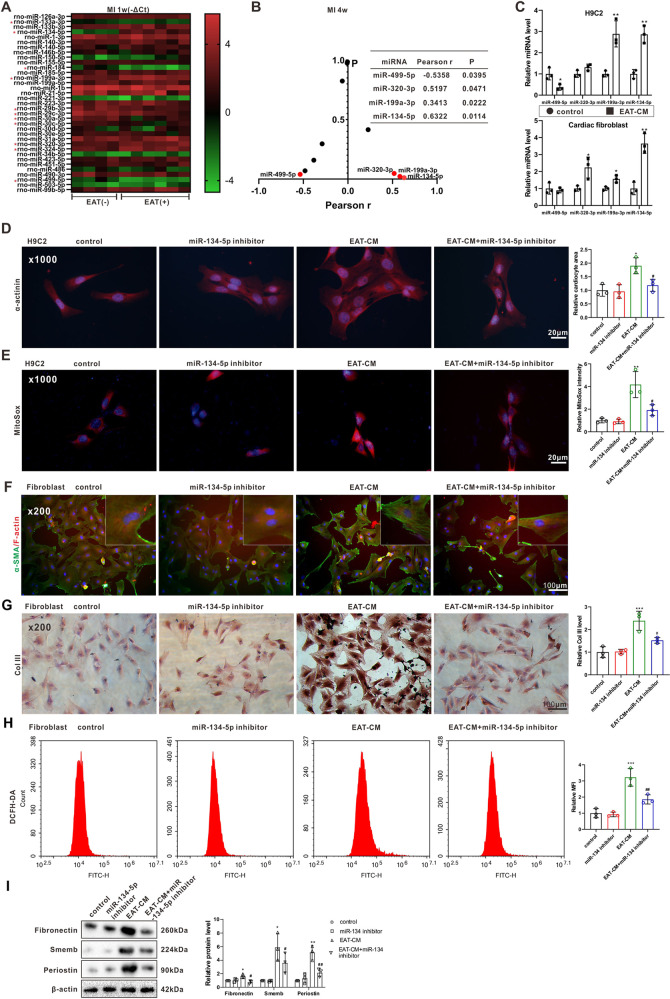
The role of miR-134-5p in cardiomyocyte hypertrophy and cardiac fibroblast activation induced by secretory products from EAT. **A** Expression profiles of 35 candidate miRNAs in the left ventricular tissues of rats with (+) or without (−) obvious EAT in MI 1-week group. **B** Pearson’s correlation coefficient (*r*) of EAT weights and expression levels of 11 differentially expressed miRNAs determined in (**A**) in MI 4-week group. **C** The expression levels of miR-499-5p, miR-320-3p, miR-199a-3p, and miR-134-5p were measured by qRT-PCR in H9C2 cells and primary rat cardiac fibroblasts cultured in DMEM-F12 medium or EAT-CM. **D**, **E** H9C2 cells were divided into control, miR-134-5p inhibitor, EAT-CM, and EAT-CM + miR-134-5p inhibitor groups. **D** Immunofluorescent staining for α-actinin (scale bar = 20 µm). Cell nuclei were counterstained with DAPI. **E** MitoSox fluorescence probe was used to observe the mitochondrial superoxide content (scale bar = 20 µm). Cell nuclei were counterstained with DAPI. **F**–**I** Primary rat cardiac fibroblasts were divided into control, miR-134-5p inhibitor, EAT-CM, and EAT-CM + miR-134-5p inhibitor groups. **F** Immunofluorescent staining for (**F**) α-SMA (green)/F-actin (red) (scale bar = 100 µm). Cell nuclei were counterstained with DAPI. **G** Immunohistochemical staining for CoI III (scale bar = 100 µm). **H** The intracellular ROS level was analyzed by flow cytometry using a DCFH-DA fluorescence probe. **I** The protein levels of fibronection, smemb, and periostin. β-actin was used as an internal control. *P < 0.05, **P < 0.01, ***P < 0.01 vs control; ^#^*P* < 0.05, ^##^*P* < 0.01 vs EAT-CM.

The GSE95855 dataset showed a total of 35 differentially expressed miRNAs (those showing more than a twofold change) in rats at 4 weeks after establishing the MI model [19]. We detected the expression levels of these candidate miRNAs in the left ventricular tissues of rats with or without obvious EAT in the MI 1-week group and found 11 miRNAs that were differentially expressed (Fig. 4A). Among them, miR-499-5p expression (*r* = −0.5358, *P* = 0.0395) was negatively correlated and miR-320-3p (*r* = 0.5197, *P* = 0.0471), miR-199a-3p (*r* = 0.3413, *P* = 0.0222), and miR-134-5p (*r* = 0.6322, *P* = 0.0114) expressions were positively correlated with the EAT weights of the rats in the MI 4-week group (Fig. 4B). Then, the expression levels of miR-499-5p, miR-320-3p, miR-199a-3p, and miR-134-5p were measured in H9C2 cells and primary rat cardiac fibroblasts cultured in EAT-CM. As shown in Fig. 4C, only miR-199a-3p and miR-134-5p were dysregulated in both H9C2 cells and primary rat cardiac fibroblasts cultured in EAT-CM. Considering the expression level of miR-134-5p demonstrated the most significant change, we focused on the role of miR-134-5p in cardiomyocyte hypertrophy and cardiac fibroblast activation induced by EAT-CM in the follow-up study. As shown in Fig. 4D, the miR-134-5p inhibitor transfection prevented the increase in cardiocyte area in H9C2 cells in response to EAT-CM culture. As shown in Fig. 4F, G, and I, the miR-134-5p inhibitor transfection suppressed the incorporation of α-SMA into F-actin stress fibers and prevented the increase in protein levels of CoI III, fibronectin, smemb, and periostin in primary rat cardiac fibroblasts cultured in EAT-CM. These findings indicating that miR-134-5p knockdown suppressed cardiomyocyte hypertrophy and cardiac fibroblast activation promoted by the secretory products from EAT. The reductions in mitochondrial superoxide content in H9C2 cells and in intracellular ROS level in primary rat cardiac fibroblasts cultured in EAT-CM + miR-134-5p inhibitor (Fig. 4E, H) indicated that miR-134-5p regulated the EAT-CM-induced cardiomyocyte hypertrophy and cardiac fibroblast activation by regulating intracellular ROS levels.

In addition, we also explored the specific secretory products from EAT that regulated miR-134-5p expression in cardiomyocytes and cardiac fibroblasts. As shown in Supplementary Fig. 2A, the miR-134-5p level was positively correlated with leptin (*r* = 0.7280, *P* < 0.05) and with activin A (*r* = 0.7084, *P* < 0.05) secreted from the EAT from rats in the MI 2-week group. Supplementations with anti-leptin and anti-activin A antibodies distinctly suppressed the increase in miR-134-5p level in H9C2 cells cultured in EAT-CM, and supplementation with anti-activin A antibody suppressed the increase in miR-134-5p level in primary rat cardiac fibroblasts cultured in EAT-CM (Supplementary Fig. 2B).

### KAT7 reversed the upregulation of intracellular ROS accumulation by miR-134-5p through promoting the transcriptions of manganese superoxide dismutase (MnSOD) and catalase

**Fig. 5:**
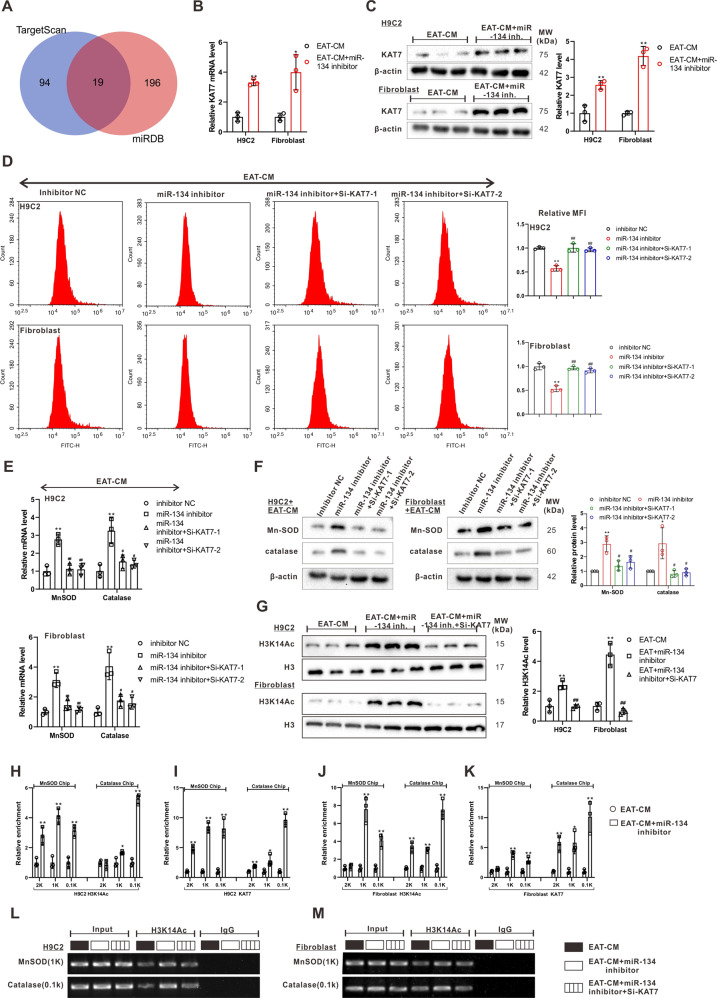
KAT7 reversed the upregulation effect of miR-134-5p on intracellular ROS accumulation by promoting the transcriptions of MnSOD and catalase. **A** Bioinformatics databases TargetScan and miRDB forecasted there were 19 common target genes of miR-134-5p. The (**B**) mRNA and (**C**) protein levels of lysine acetyltransferase 7 (KAT7) in H9C2 cells and primary rat cardiac fibroblasts treated with EAT-CM or EAT-CM + miR-134-5p inhibitor were measured by qRT-PCR and western blot, respectively. β-actin was used as an internal control. **D**–**F** H9C2 cells and primary rat cardiac fibroblasts were divided into four groups: miR-134-5p inhibitor, the negative control of miR-134-5p inhibitor (inhibitor NC), miR-134-5p inhibitor + si- lysine acetyltransferase 7 (KAT7)-1, miR-134 -5p inhibitor + si-KAT7-2. All the cells were cultured in EAT-CM. **D** The intracellular ROS level was analyzed by flow cytometry using a DCFH-DA fluorescence probe. The (**E**) mRNA and **F** protein levels of manganese superoxide dismutase (MnSOD) and catalase. **G** H9C2 cells and primary rat cardiac fibroblasts were divided into three groups: EAT-CM, EAT-CM + miR-134-5p inhibitor, EAT-CM + miR-134-5p inhibitor + si-KAT7. Histone H3K14 acetylation (H3K14Ac) level of cells was analyzed using Western blot. H3 was used as an internal control. **H**–**K** H9C2 and primary rat cardiac fibroblasts were divided into EAT-CM and EAT-CM + miR-134-5p inhibitor groups. **H**, **J** The H3K14Ac levels of 2 kb, 1 kb, and 0.1 kb upstream of transcription start site (TSS) of MnSOD/catalase gene were measured by chromatin immunoprecipitation (CHIP)-PCR. **I**, **K** The combination between KAT7 and 2 kb, 1 kb, and 0.1 kb upstream of TSS of MnSOD/ catalase gene was measured by CHIP-PCR. **L**, **M** H9C2 cells and primary rat cardiac fibroblasts were divided into three groups: EAT-CM, EAT-CM + miR-134-5p inhibitor, EAT-CM + miR-134-5p inhibitor + si-KAT7. The H3K14Ac levels of 1 kb upstream of TSS of MnSOD gene and 0.1 kb upstream of TSS of catalase gene were measured by CHIP-PCR. **B**, **C**, **H**–**K** *P < 0.05, **P < 0.01 vs EAT-CM. **D**–**F** **P < 0.01 vs inhibitor NC; ^#^*P* < 0.05, ^##^*P* < 0.01 vs miR-134-5p inhibitor. **G** **P < 0.01 vs EAT-CM; ^##^*P* < 0.01 vs EAT-CM + miR-134-5p inhibitor.

The miRNAs are well known to exert their functions by binding the 3’UTR of target genes [20, 21]. The bioinformatics databases TargetScan and miRDB forecasted 19 common target genes of miR-134–5p (Fig. 5A). To determine the specific target genes of miR-134-5p in H9C2 cells and primary rat cardiac fibroblasts, the mRNA levels of 19 candidate genes were measured in H9C2 cells and primary rat cardiac fibroblasts cultured in EAT-CM or EAT-CM + miR-134-5p inhibitor. As shown in Supplementary Fig. 3A and Fig. 5B, the mRNA levels of protein phosphatase 1 regulatory subunit 7 (PPP1R7), lysine acetyltransferase 7 (KAT7), and ABL proto-oncogene 2 (ABl2) were negatively regulated by the miR-134-5p inhibitor in both H9C2 cells and primary rat cardiac fibroblasts cultured in EAT-CM. Meanwhile, the results of western blot showed upregulation of the protein levels of KAT7 (Fig. 5C) and ABl2 (Supplementary Fig. 3B) by miR-134-5p inhibitor in both H9C2 cells and primary rat cardiac fibroblasts cultured in EAT-CM (PPP1R7 was not studied due to a lack of a commercially available antibody). Afterward, si-KAT7 or si-ABl2 were transfected into H9C2 cells and primary rat cardiac fibroblasts cultured in EAT-CM. As shown in Fig. 5D and Supplementary Fig. 3C, si-KAT7, but not si-ABl2, effectively abrogated the downregulatory effect of the miR-134-5p inhibitor on intracellular ROS levels in both H9C2 cells and primary rat cardiac fibroblasts cultured in EAT-CM. Meanwhile, correlation analysis demonstrated a negative correlation between the KAT7 mRNA level and the miR-134-5p level in the left ventricular tissues of rats in the MI 4-week group (*r* = −0.6408, *P* = 0.0101) (Supplementary Fig. 4A). The dual-luciferase reporter gene assay showed that the miR-134-5p mimic significantly decreased the relative light unit of pmirGLO-WT KAT7 3’UTR (Supplementary Fig. 4B). Overall, these findings confirmed that KAT7 was the miR-134-5p target gene responsible for regulating ROS level in H9C2 cells and primary rat cardiac fibroblasts cultured in EAT-CM.

In addition, the miR-134-5p inhibitor apparently boosted the mRNA and protein levels of MnSOD and catalase, two enzymes essential for ROS scavenging [22], in both H9C2 cells and primary rat cardiac fibroblasts cultured in EAT-CM, and this effect was reversed by si-KAT7 (Fig. 5E, F). As reported, KAT7 (also known as HBO1) is a member of the MYST family of histone acetyltransferases (HATs) and functions in the acetylation of histone H3K14 (H3K14Ac) [23]. The western blot analysis revealed that si-KAT7 transfection inhibited the increase in H3K14Ac level in H9C2 cells and primary rat cardiac fibroblasts treated with miR-134-5p inhibitor + EAT-CM (Fig. 5G). Therefore, we investigated whether KAT7 knockdown decreased the transcription of MnSOD and catalase by suppressing the increase in H3K14Ac induced by the miR-134-5p inhibitor. As shown in Fig. 5H–K, the miR-134-5p inhibitor upregulated the H3K14Ac level of the 1 kb and 0.1 kb upstream of the TSS of MnSOD and catalase genes and reinforced the combination between KAT7 and 1 kb and 0.1 kb upstream of the TSS of MnSOD and catalase genes in H9C2 cells and primary rat cardiac fibroblasts cultured in EAT-CM. KAT7 knockdown suppressed the H3K14Ac levels at 1 kb upstream of the TSS of the MnSOD gene and at 0.1 kb upstream of the TSS of the catalase gene in both H9C2 cells and primary rat cardiac fibroblasts cultured in EAT-CM (Fig. 5L, M). These findings indicated that miR-134-5p suppressed KAT7 expression, removed KAT7-induced H3K14Ac of MnSOD and catalase promoters, thereby promoting ROS accumulation in H9C2 cells and primary rat cardiac fibroblasts cultured in EAT-CM.

### Knockdown of miR-134 alleviated adverse myocardial remodeling in the rat model of MI

**Fig. 6:**
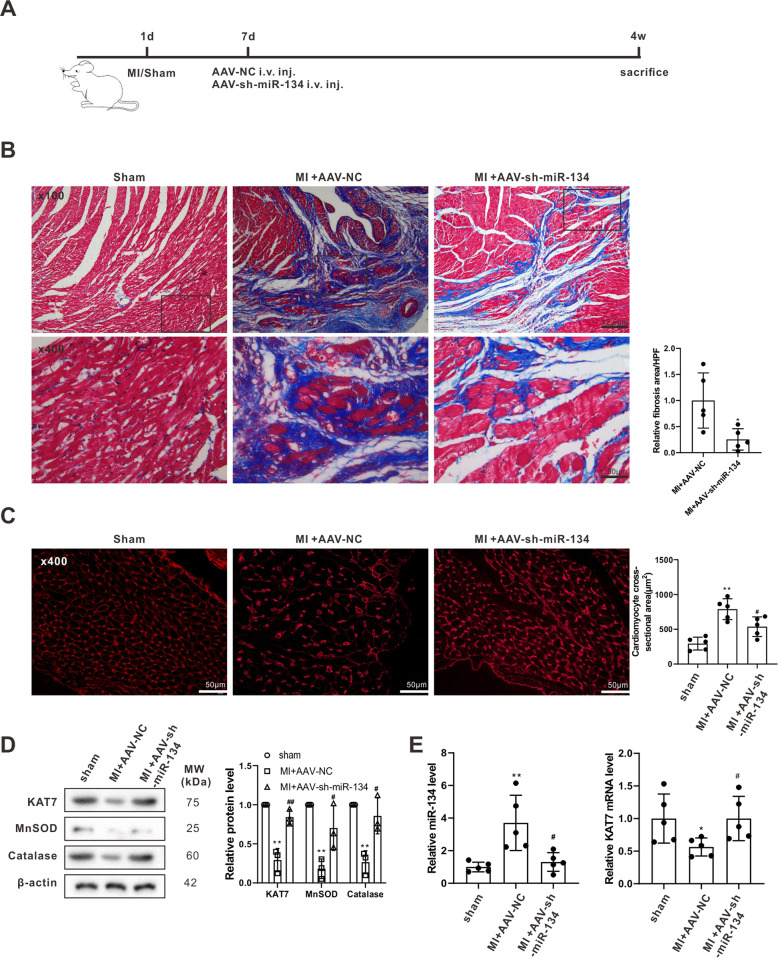
miR-134 knockdown alleviated adverse myocardial remodeling in the rat model of MI. **A** The experimental protocols of sham (*n* = 10), MI + adeno-associated virus (AAV)-sh-miR-134-5p (*n* = 10), and MI + the negative control of AAV-sh-miR-134-5p (AAV-NC) (*n* = 10). **B** Myocardial fibrosis was examined using Masson’s trichrome staining. **C** Cardiomyocyte cross-sectional area was examined using WGA staining (scale bar = 50 µm). **D** The protein levels of KAT7, MnSOD, and catalase in the left ventricular tissues of rats in each group. **E** The miR-134-5p level and KAT7 mRNA level in the left ventricular tissues of rats in each group. **B** *P < 0.05 vs MI + AAV-NC. **C**–**E** *P < 0.05, **P < 0.01 vs sham; ^#^*P* < 0.5 vs MI + AAV-NC.

The experimental protocol for investigating the in vivo evaluation of the effect of miR-134-5p on the adverse myocardial remodeling after MI in the sham, MI + AAV-NC, and MI + AAV-sh-miR-134-5p groups is presented in Fig. 6A. The suppression of the miR-134-5p levels in the left ventricular tissues of rats in MI + AAV-sh-miR-134-5p groups confirmed that intravenous injection with AAV-sh-miR-134-5p successfully silenced miR-134-5p expression (Fig. 6E). As shown in Fig. 6B, C, compared with AAV-NC, AAV-sh-miR-134-5p prominently alleviated myocardial fibrosis and decreased cardiomyocyte cross-sectional areas in rats with MI, without affecting EAT weight (data not shown). Besides, elevations in the expression levels of KAT7, MnSOD, and catalase were observed in the left ventricular tissues of rats in the MI + AAV-sh-miR-134-5p groups (Fig. 6D, E), indicating that the therapeutic effect of miR-134-5p in preventing the adverse myocardial remodeling after MI was associated with KAT7-mediated ROS degradation.

## Discussion

This study provided the first direct evidence for the potential role of EAT and its secretory products in the adverse myocardial remodeling that occurred after MI. In this study, obvious EAT was observed in the rat model of MI, and the in vitro data showed that EAT-CM increased ROS level through a miR-134-5p/KAT7/MnSOD/catalase axis, thereby increasing cardiomyocyte size and activating cardiac fibroblast. The in vivo data confirmed that miR-134-5p knockdown effectively limited adverse myocardial remodeling in the rat model of MI and was manifested as less myocardial hypertrophy and fibrosis.

In the last 20 years, researchers have gradually realized the essential role of adipose tissues as an endocrine organ with multiple metabolic functions [24]. EAT, as a component of the visceral adipose tissues around the heart, is intricately related to cardiovascular health [25]. Under normal physiologic conditions, EAT exerts its cardioprotective effect by providing mechanical protection, by supplying energy to the myocardium, and by secreting anti-inflammatory bioactive molecules. However, under pathologic conditions, the EAT enlarges and causes local cardiac pathology by secreting pro-inflammatory bioactive molecules through vasocrine or paracrine pathways [26]. A prospective investigation by Erkan et al. [27] revealed that EAT thickness was positively correlated with the extent and complexity of coronary artery disease (CAD). Research by Mancio et al. [28] also showed that EAT volume might contribute to coronary calcification in the advanced stage of CAD. Gruzdeva et al. [5] also found that EAT thickness in MI patients was upregulated and positively associated with the onset of myocardial fibrosis. Consistent with these previous studies, obvious EAT was observed in this study in the rat model of MI one week after LAD ligation. A positive correlation was also observed between EAT weights and cardiomyocyte size/fibrosis areas in the rats in the MI 2-week and 4-week groups. The exposure to EAT-CM significantly increased the cell surface area of H9C2 cells and facilitated the activation of primary rat cardiac fibroblasts. These results indicated that EAT and its secretory products contributed to myocardial hypertrophy and fibrosis after MI. The research of Venteclef et al. [29] also supports our findings, as they found that EAT promoted fibrosis of rat atria by producing activin A. Nevertheless, the specific mechanism by which EAT and its secretory products induce myocardial hypertrophy and fibrosis after MI remains obscure.

Through the detection of mitochondrial superoxide content in H9C2 cells and the intracellular ROS level in primary rat cardiac fibroblasts, we found that the enlarged cardiomyocytes and enhanced cardiac fibroblast activation induced by EAT-CM were accompanied by the upregulation of ROS level. The promoting effect of superabundant ROS on adverse myocardial remodeling has been widely proven. A previous study has shown that sirtuin 4 boosted the hypertrophic growth of cardiomyocytes and the generation of myocardial fibrosis by elevating ROS accumulation in a mouse model of pathological cardiac hypertrophy [30]. Other work has shown that melatonin can prevent adverse myocardial remodeling by relieving mitochondrial impairment and inhibiting ROS production in the mouse model of MI [31]. In our study, the ROS scavenger NAC eliminated the regulatory effects of EAT-CM on cardiomyocyte size and cardiac fibroblast activation, indicating that the EAT-CM effects are functionally dependent on ROS level.

Then, the molecular mechanism by which EAT-CM upregulated ROS accumulation was explored. miR-134-5p was a differentially expressed miRNA in MI, which was firstly reported by Wang et al. [32]. In recent years, the biological role of miR-134-5p in MI has been verified. Li et al. [33] reported that miR-134-5p was highly expressed in infarction tissues in a mouse model of MI and that miR-134-5p knockdown enhanced myocardial angiogenesis and suppressed cardiomyocyte apoptosis. The elevation of miR-134-5p in H9C2 cells and primary rat cardiac fibroblasts cultured in EAT-CM observed in our study indicated that the high expression of miR-134-5p in infarction tissues after MI reported by Li et al. [33] might be regulated by enlarged EAT. In addition, we also observed that miR-134-5p knockdown reversed the cardiomyocyte hypertrophy and cardiac fibroblast activation induced by EAT-CM by suppression of ROS accumulation. The following experiments determined the inhibitory effect of miR-134-5p on ROS accumulation was dependent on KAT7, a HAT responsible for the H3K14Ac of MnSOD and catalase. During the initial phase of ROS production, molecular oxygen is converted to highly reactive superoxide. MnSOD is the key enzyme that converts superoxide to less reactive hydrogen peroxide (H_2_O_2_), which is then converted to harmless H_2_O by catalase or glutathione peroxidase [34]. As previously reported, H3K14Ac facilitates RNA polymerase II-mediated transcript elongation to maintain the overexpression of target genes [35]. Through its suppression of KAT7 expression by binding to the 3’ UTR, miR-134-5p inhibited the KAT7-induced H3K14Ac of MnSOD and catalase, thereby decreasing the expressions of MnSOD and catalase and promoting ROS accumulation. In parallel with our study, Aksu-Menges et al. [36] identified miR-134-5p as a commonly increased miRNA related to mitochondrial damage in patients with muscular dystrophy, thereby revealing a potential link between miR-134-5p and mitochondrial dysfunction.

Atrial fibrillation (AF) is one of the cardiac arrhythmia diseases that seriously threaten human health globally [37]. One clinically recognized independent risk factor for new-onset AF is MI, as this promotes the development of adverse myocardial remodeling [38]. Statistically, adverse myocardial remodeling occurs after MI in 10% of patients, who then show pathological myocardial hypertrophy and fibrosis [39], as well as, increased AF susceptibility [40, 41]. Since our study showed that miR-134-5p knockdown relieved adverse myocardial remodeling post MI, we speculated that miR-134-5p may also have a potential role in AF progression.

In general, our study clarified a new pathological mechanism involving an EAT/miRNA axis that explains the adverse myocardial remodeling occurring after MI and provides novel intervention targets for the clinical prevention and treatment of this adverse aftereffect of MI.

## Supplementary information

supplementary material

Supplementary

Supplementary Figure 1

Supplementary Figure 2

Supplementary Figure 3

Supplementary Figure 4
