# Supplementary material for: Secretory products from epicardial adipose tissue induce adverse myocardial remodeling after myocardial infarction by promoting reactive oxygen species accumulation
Source: Cell Death Dis. 2021 Sep 13;12(9):848. doi: 10.1038/s41419-021-04111-x (PMC8438091; doi:10.1038/s41419-021-04111-x)
Supplement: Supplementary file 1 — supplementary material [file 41419_2021_4111_MOESM1_ESM.docx]

**Triphenyltetrazolium chloride (TTC) staining**

Hearts collected from rats in the control and MI 1 week groups (*n* = 3) were washed with saline solution and sectioned into 2 mm thick transverse slices from apex to base. The slices were immersed in a 1% TTC solution (Solarbio Technology Co., Ltd, Beijing, China) for 20 min and then followed photographed.

**Histological staining**

Hematoxylin-eosin (H&E) staining, wheat germ agglutinin (WGA) staining, and Masson’ trichrome staining were carried out with the cardiac tissues at the location indicated by the dotted line in Figure 1 A. Tissues were fixed with 4% paraformaldehyde, embedded in paraffin, and sectioned into 5 mm thick sections. H&E staining was performed using the Hematoxylin-Eosin/HE Staining Kit (Solarbio Technology Co., Ltd) according to the manufacturer’s instruction. Masson's trichrome staining was performed using a Masson’s Trichrome Stain Kit (Solarbio Technology Co., Ltd) according to the manufacturer’s instruction. WGA staining was conducted by incubating sections with 5 µg/mL WGA (Thermo Fisher Scientific, Waltham, MA, USA) for 10 min at room temperature and then viewing and photographing with a fluorescence microscope.

**Dihydroethidium (DHE) staining**

The accumulation of ROS in the cardiac tissues of rats in each group was evaluated using a DHE staining kit (Bio Lebo Technology Co., Ltd., Beijing, China). Cryosections (4 μm) were prepared using the fresh cardiac tissues of rats and incubated with 100 μl staining working solution containing DHE probe for 40 min at 37℃. The stained sections were then washed with PBS and photographed with a fluorescence microscope.

**Primary rat cardiac fibroblast isolation**

Adult rat cardiac fibroblasts were isolated from 10-week old SD rats. Briefly, each rat was anesthetized, and the heart was excised and washed with cold saline solution. Then, the heart was minced into small pieces, followed by digestion with collagenase II (Sigma-Aldrich, St. Louis, MO, USA). After centrifuging at 2000 rpm for 5 min, the pellet was suspended in DMEM-F12 medium (Thermo Fisher Scientific) containing 10% fetal bovine serum (FBS; Thermo Fisher Scientific). After 2 h, the non-adherent cells were removed and the cardiac fibroblasts attached to plates were cultured in DMEM-F12 medium supplemented with 10% FBS, 33 µmol/l biotin, 17 µmol/l D-pantothenate, and antibiotics at 37℃. To evaluate the effect of EAT and miR-134-5p on fibroblast activation, primary rat cardiac fibroblasts were seed on a 6-well CytoSoft® plate with stiffness of 8 kPA (Advanced Biomatrix, San Diego, CA, USA) to obtain quiescent fibroblasts.

**Immunofluorescence staining**

H9C2 cells and primary rat cardiac fibroblasts were seeded on 6-well plates and subjected to the desired experimental protocol. For immunofluorescence staining, cells were fixed with 4% paraformaldehyde and then blocked with 5% bovine serum albumin (BSA). The H9C2 cells were incubated with anti-α-actinin antibody (Thermo Fisher Scientific) and the primary rat cardiac fibroblasts were incubated with anti-alpha-smooth muscle actin (α-SMA) (Abcam, Cambridge, UK) + Filamentous actin (F-actin) (Thermo Fisher Scientific) antibodies at 4℃ overnight. On the second day, the H9C2 cells were incubated with goat anti-Rabbit IgG-H&L (Alexa Fluor® 647) (Abcam), and the primary rat cardiac fibroblasts were incubated with Goat anti-Rabbit IgG-H&L (Alexa Fluor® 488) Abcam) or/and Goat Anti-Mouse IgG H&L (Alexa Fluor® 647) (Abcam) for 1 h at 37℃. The nuclei were counterstained with DAPI and the stained cells were photographed with a fluorescence microscope.

[**Immunohistochemical**](javascript:;) **staining**

Primary rat cardiac fibroblasts subjected to the desired experimental protocol were fixed with 4% paraformaldehyde and blocked with 5% BSA. Then, cells were incubated with anti-collagen Ⅲ (CoI Ⅲ) (Abcam) at 4℃ overnight. On the second day, cells were orderly incubated with Goat anti-Rabbit IgG and SABC-AP (Boster Biological Technology Co., Ltd., Wuhan, China) for 30 min at 37℃, followed by the incubation with BCIP/NBT (Boster Biological Technology Co., Ltd). Nuclei were counterstained with [hematoxylin](javascript:;) and the stained cells were photographed with a microscope.

**Mitochondrial superoxide content measurement**

H9C2 cells were seeded on coverslips in 24-well plates and cultured in DMEM-F12 medium, EAT-CM, or EAT-CM+N-acetyl-cysteine (NAC). Three days later, the cells in each well were incubated with 1 ml 5 µM MitoSox red mitochondrial superoxide indicator (Yeasen Biotechnology Co., Ltd, Shanghai, China) for 10 min in the dark. The cells were washed with Hanks Balanced Salt Solution (HBSS), and the MitoSox intensity was detected by fluorescence microscopy.

**Intracellular ROS measurement**

H9C2 cells and primary rat cardiac fibroblasts were seeded in 6-well plates and subjected to the desired experimental protocol. The original medium was replaced with a serum-free medium containing 10 µM DCFH-DA fluorescence probe (Beyotime Biotechnology Co., Ltd, Shanghai, China). After 20 min, cells were washed with HBSS, and the DCFH-DA intensity was detected by flow cytometry.

**Bioinformatics analysis**

A miRNA expression dataset GSE95855 containing cardiac tissue samples from rats with MI (*n* = 3) and rats with the sham operation (*n* = 3) were downloaded. The differential screening was performed by setting fold change > 2 or fold change < 0.5 as thresholds and 35 candidate miRNAs were determined.

**Cell transfection**

The miR-134-5p inhibitor, miR-134-5p mimic, si-RNA targeting lysine acetyltransferase 7 (si-KAT7-1 and si-KAT7-2), and their negative controls (inhibitor NC and NC) were all synthesized by GenePharma (Shanghai, China). H9C2 cells and primary rat cardiac fibroblasts were seeded in 6-well plates at a density of 1 $\text{×}$ 10^6^ cells/well. For transfection, 250 µl Opti-MEM medium containing 2500 ng miR-134-5p inhibitor, miR-134-5p mimic, si-KAT7, inhibitor NC, or NC, and 7.5 µl Lipofectamine™ 3000 reagent (Thermo Fisher Scientific) was added to each well and incubated with cells for 48 h.

**Western blot**

Protein samples were isolated from H9C2 cells, primary rat cardiac fibroblasts, and the rat left ventricular tissues using RIPA buffer (Solarbio Technology Co., Ltd). Total histone samples were isolated from H9C2 cells and primary rat cardiac fibroblasts using an EpiQuik Total Histone Extraction Kit (Epigentek, Farmingdale, NY, USA). An equal amount of protein samples were subjected to 10% SDA-PAGE, followed by transferring to a PVDF membrane. After blocking with 5% BSA, the membranes were incubated with the following primary antibodies: anti-fibronection (ab268020, Abcam), anti-smemb (Thermo Fisher Scientific), anti-periostin (Abcam), anti-KAT7 (Abcam), anti-manganese superoxide dismutase (MnSOD) (Abcam), anti-catalase (Abcam), anti-histone H3 (acetyl K14) (Abcam), and anti-ABL proto-oncogene 2 (ABl2) (Abcam) at 4℃. The next day, the membranes were incubated with goat-anti-rabbit IgG H&L (HRP) (Abcam) for 1 h. The membranes were visualized using the ChemiDoc MP Imaging System (Bio-Rad, Hercules, CA, USA) with the assistance of ECL Western Blotting Substrate (Solarbio Technology Co., Ltd).

**qRT-PCR**

miRNAs /total RNA samples were isolated from H9C2 cells, primary rat cardiac fibroblasts, and the rat left ventricular tissues using the miRNeasy Tissue/Cells Advanced Kits (Qiagen, Duesseldorf, Germany)/RNeasy Kits (Qiagen) and reversely transcribed into cDNA using the miScript II RT Kit (Qiagen). The qRT-PCR was performed using miScript SYBR® Green PCR Kit (Qiagen) on a real-time PCR system (Applied Biosystems, Carlsbad, CA, USA). The expression levels of miR-499-5p, miR-320-3p, miR-199a-3p, and miR-134-5p and the mRNA levels of MnSOD, catalase, and KAT7 were calculated using the 2^-ΔΔCT^ method.

**Chromatin immunoprecipitation (CHIP)**

The histone H3K14 acetylation levels of MnSOD/catalase and the combination between KAT7 and the MnSOD/catalase gene promoters were assessed using CHIP assay. Briefly, 5$\text{×}$10^6^ H9C2 cells or primary rat cardiac fibroblasts were harvested and fixed with 1% formaldehyde. The cells were then broken open by sonication and centrifuged. The supernatant was collected and incubated overnight with beads coated with anti-histone H3 (acetyl K14) antibody Abcam) or with anti-KAT7 antibody (Cell Signaling Technology, Danvers, MA, USA). The endogenous DNA-protein complex was then eluted and subjected to reverse crosslinking. The enrichment of MnSOD/catalase gene promoter binding by anti-histone H3 (acetyl K14) antibody or anti-KAT7 antibody was determined by PCR using the specific primers 2 kb, 1 kb, and 0.1 kb upstream of the transcription start site (TSS) of the MnSOD/catalase gene.

**Dual-luciferase reporter assay**

The interplay between KAT7 and miR-134-5p was evaluated using the dual-luciferase reporter assay. Before the assay, the sequence of the wild type (WT) KAT7 mRNA 3’ untranslated region (UTR) or the mutant type (Mut) KAT7 mRNA 3’UTR was subcloned into pmirGLO plasmids (Promega, Madison, WI, USA). The miR-134-5p mimic/NC and pmirGLO-WT KAT7 3’UTR/pmirGLO-Mut KAT7 3’UTR were cotransfected into HEK293T cells. Two days after transfection, the relative light units of pmirGLO-WT KAT7 3’UTR and pmirGLO-Mut KAT7 3’UTR were measured with the dual-luciferase reporter assay system (Promega).

**Statistical analysis**

All statistical analyses were performed using GraphPad Prism 7.0 (GraphPad, La Jolla, CA, USA). The results were expressed as the means ± standard deviations . The statistical significance of the differences between the two experimental groups was evaluated using a Student’s t-test. Pearson’s correlation coefficient (r) was used to determine the correlation between variables. A value of *P*<0.05 was considered statistically significant.
