# Supplementary material for: Secretory products from epicardial adipose tissue induce adverse myocardial remodeling after myocardial infarction by promoting reactive oxygen species accumulation
Source: Cell Death Dis. 2021 Sep 13;12(9):848. doi: 10.1038/s41419-021-04111-x (PMC8438091; doi:10.1038/s41419-021-04111-x)
Supplement: Supplementary file 2 — Supplementary [file 41419_2021_4111_MOESM2_ESM.docx]

**Supplementary Figure 1** The representative images of (A) H&E (scale bar = 1 mm; triangles: EAT ), (B) Masson’s trichrome (scale bar = 1 mm), and (C) Dihydroethidium staining (scale bar = 50 μm) performed on cardiac tissues of rats in control, MI 1 week, MI 2 week, and MI 4 week groups. The EAT weights were positively correlated with (D and E) cardiomyocyte size and (F and G) myocardial fibrosis areas of rats in MI 2 week and MI 4 week groups.

**Supplementary Figure 2** (A) Ten EAT samples of rats in the MI 2 week group were cultured separately. Three days later, the contents of leptin, TGF-β1, activin A, and VEGF in culture medium were measured using ELISA. Then, the correlation analysis was performed to determine the correlation between the miR-134-5p level and the contents of leptin, TGF-β1, activin A, and VEGF. (B) EAT-CM was collected from EAT of rats in MI 2 week and 4 week groups, respectively. H9C2 cells and primary rat cardiac fibroblasts were cultured in DMEM-F12 medium, 2 w /4 w EAT-CM, or 2 w /4 w EAT-CM supplemented with anti-leptin or anti-activin A antibody, and the miR-134-5p level was measured. **P*<0.05, ***P*<0.01.

**Supplementary Figure 3** (A) The expression profile of 19 common target genes of miR-134-5p were measured in H9C2 cells and primary rat cardiac fibroblasts treated with EAT-CM or EAT-CM+miR-134-5p inhibitor. (B) The protein level of ABL proto-oncogene 2 (ABl2) in H9C2 cells and primary rat cardiac fibroblasts treated with EAT-CM or EAT-CM+miR-134-5p inhibitor. (C) H9C2 cells and primary rat cardiac fibroblasts were divided into four groups: miR-134-5p inhibitor, inhibitor NC, miR-134-5p inhibitor+si-ABl2-1, miR-134-5p inhibitor+si-ABl2-2. All the cells were cultured in EAT-CM. The intracellular ROS level was analyzed by flow cytometry using a DCFH-DA fluorescence probe.

**Supplementary Figure 4** (A) The correlation analysis of the miR-134-5p level and KAT7 mRNA level in the left ventricular tissues of rats in the MI 4 week group. (B) Dual-luciferase reporter gene assay was performed to determine the interplay between KAT7 and miR-134-5p. rno= Rattus norvegicus, mmu= Mus musculus, homo= Homo sapiens. RLU= relative light unit. **P*<0.05 vs pmirGLO-WT KAT7 3’UTR + mimic NC.
