# Supplementary figures and images for: Secretory products from epicardial adipose tissue induce adverse myocardial remodeling after myocardial infarction by promoting reactive oxygen species accumulation
Source: Cell Death Dis. 2021 Sep 13;12(9):848. doi: 10.1038/s41419-021-04111-x (PMC8438091; doi:10.1038/s41419-021-04111-x)

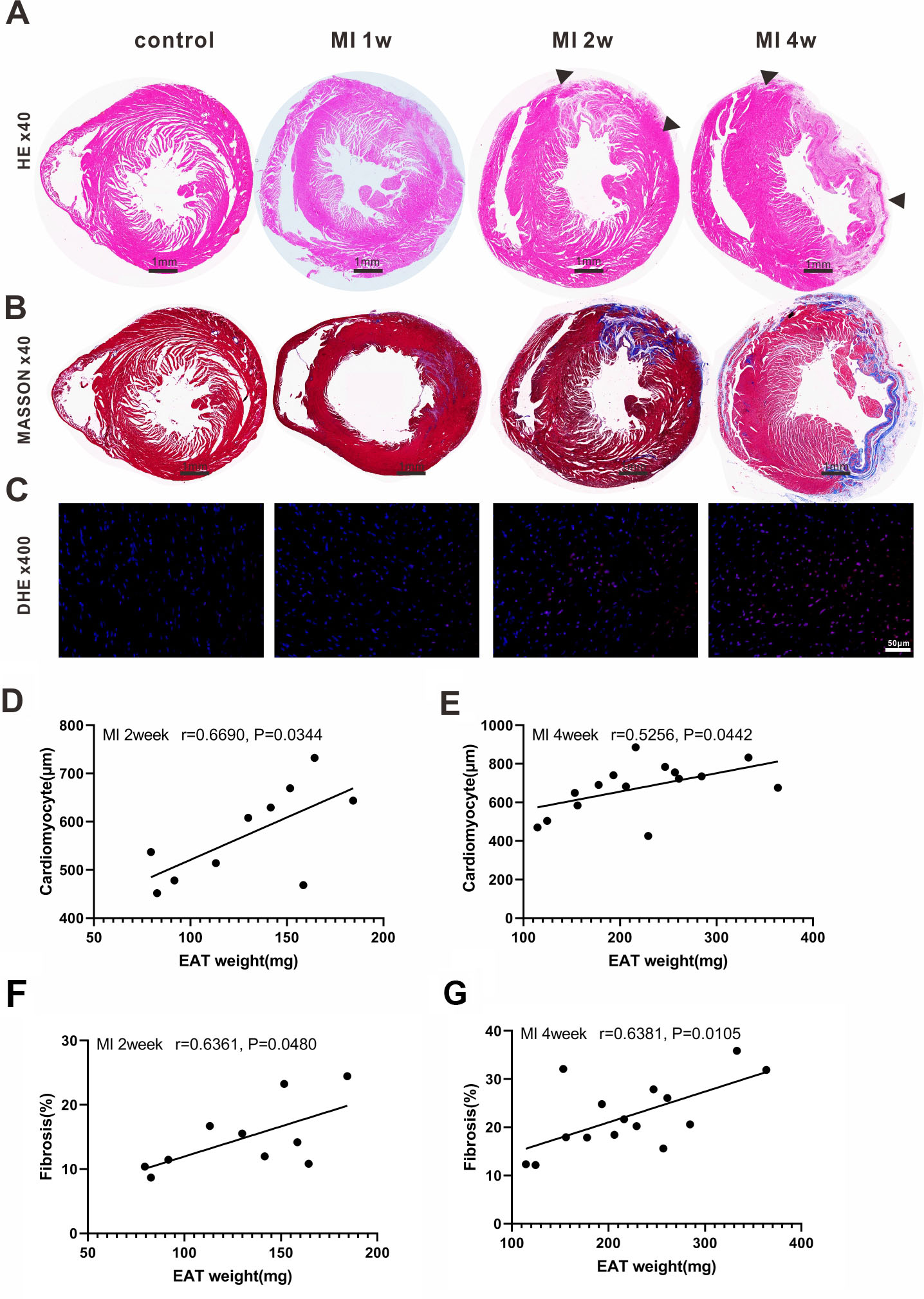

Supplement: Supplementary file 3 — Supplementary Figure 1 [file 41419_2021_4111_MOESM3_ESM.tif]

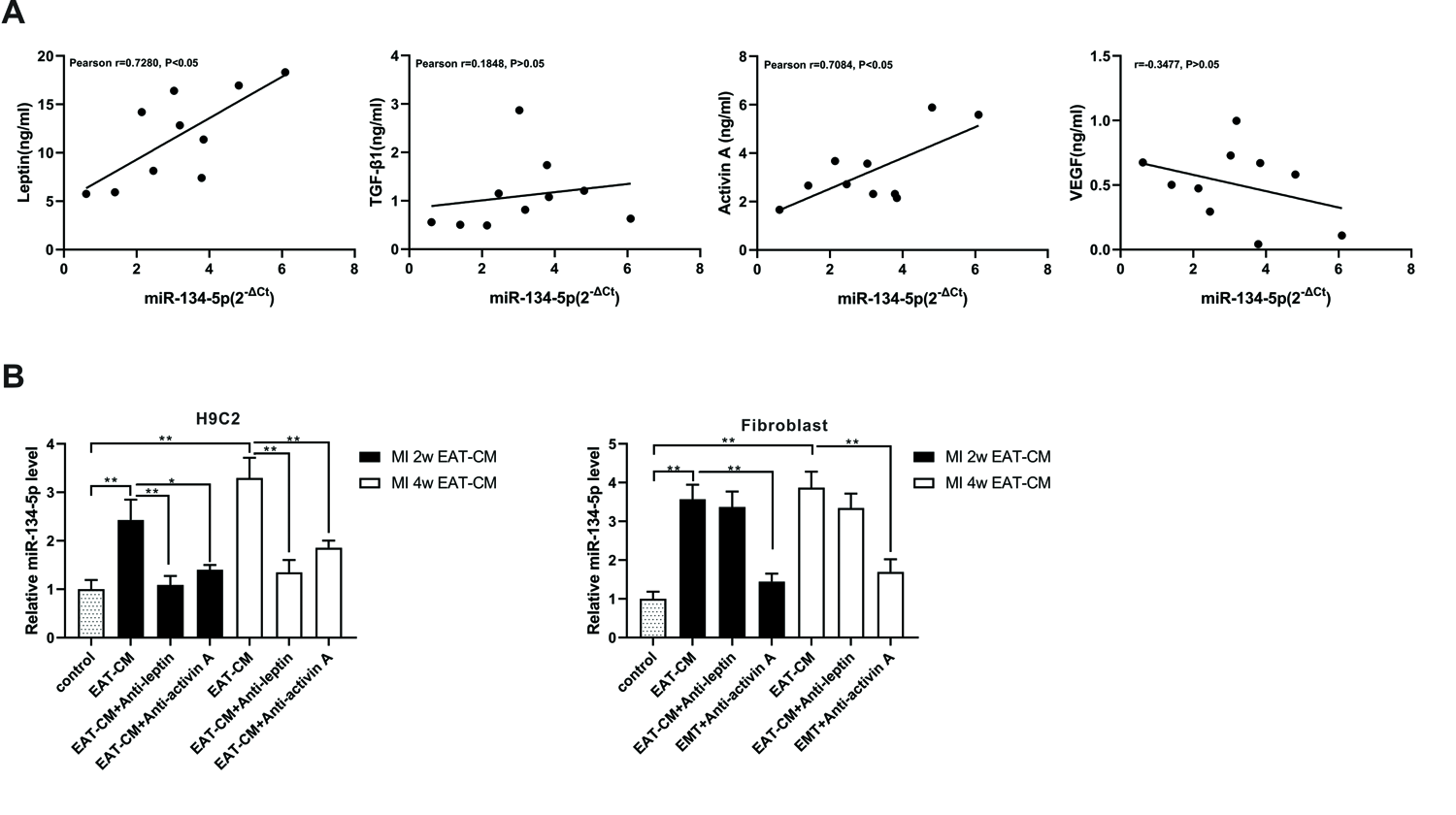

Supplement: Supplementary file 4 — Supplementary Figure 2 [file 41419_2021_4111_MOESM4_ESM.tif]

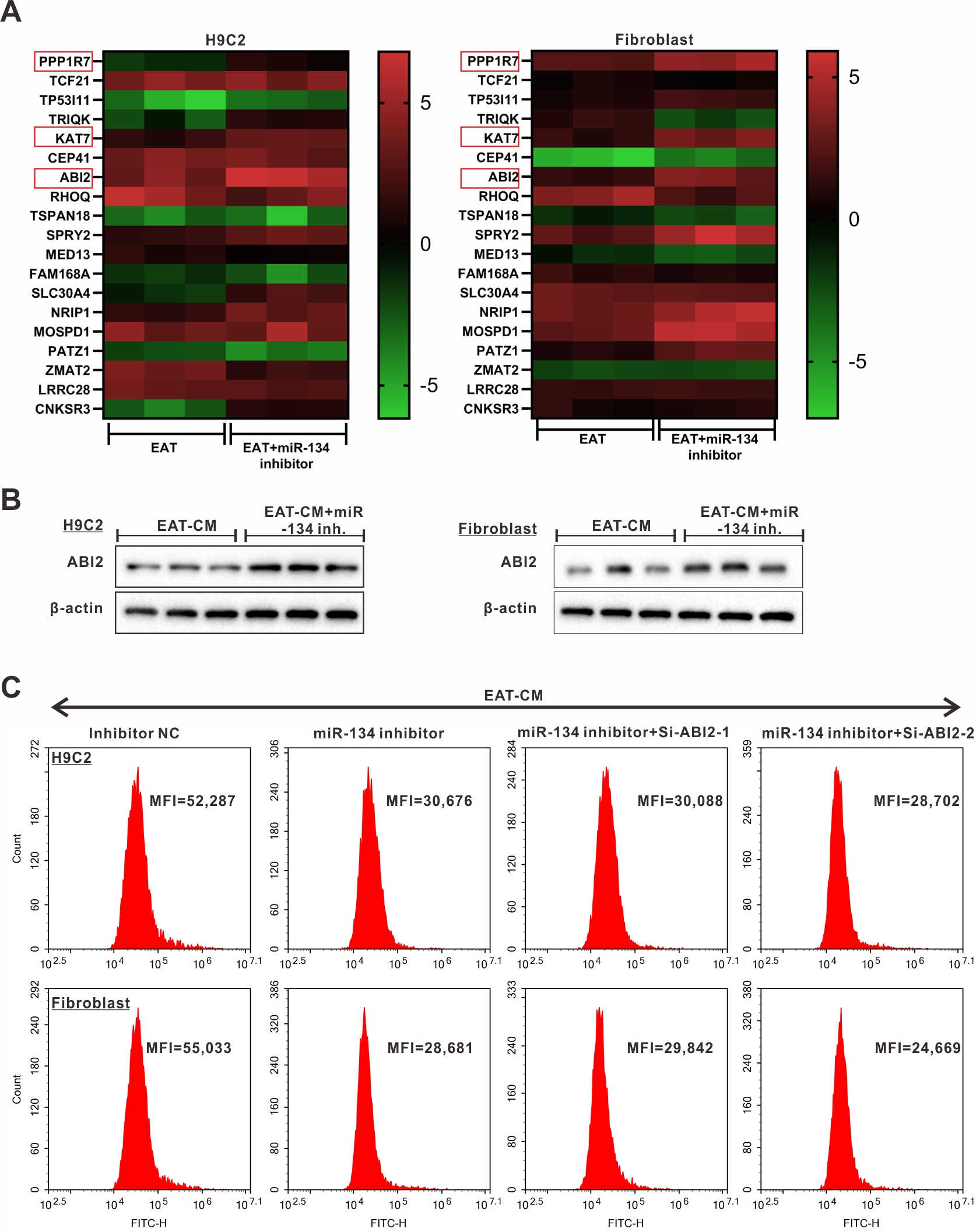

Supplement: Supplementary file 5 — Supplementary Figure 3 [file 41419_2021_4111_MOESM5_ESM.tif]

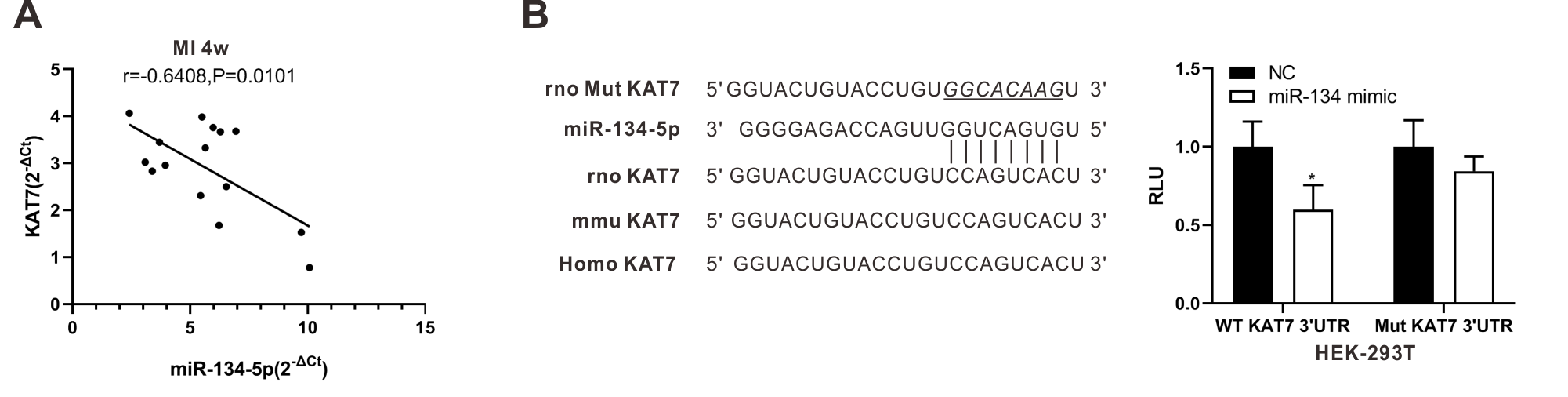

Supplement: Supplementary file 6 — Supplementary Figure 4 [file 41419_2021_4111_MOESM6_ESM.tif]
